# MicroRNA-197 controls ADAM10 expression to mediate MeCP2’s role in the differentiation of neuronal progenitors

**DOI:** 10.1038/s41418-018-0257-6

**Published:** 2018-12-18

**Authors:** Yu-Meng Wang, Yu-Fang Zheng, Si-Yu Yang, Zhang-Min Yang, Lin-Na Zhang, Yan-Qin He, Xiao-Hong Gong, Dong Liu, Richard H. Finnell, Zi-Long Qiu, Ya-Song Du, Hong-Yan Wang

**Affiliations:** 1 Institute of Developmental Biology & Molecular Medicine, School of Life Sciences, Fudan University, 200433 Shanghai, China; 2 Obstetrics and Gynecology Hospital, Institute of Reproduction and Development, State Key Laboratory of Genetic Engineering at School of Life Sciences, Fudan University, 200011 Shanghai, China; 3 Key Laboratory of Reproduction Regulation of NPFPC, Collaborative Innovation Center of Genetics and Development, Fudan University, 200032 Shanghai, China; 4 Department of Biochemistry and Molecular Biology, College of life Sciences, Shaanxi Normal University, 710062 Xi’an, China; 5 Shanghai Mental Health Center, Shanghai Jiaotong University, 200030 Shanghai, China; 6 Co-innovation Center of Neuroregeneration, Jiangsu Key Laboratory of Neuroregeneration, Nantong University, 226001 Nantong, Jiangsu China; 7 Departments of Molecular and Cellular Biology and Medicine, Baylor College of Medicine, Houston, TX 77030 USA; 8 Collaborative Innovation Center for Genetics & Development, School of Life Sciences, Fudan University, 200438 Shanghai, China; 9 Institute of Neuroscience, Shanghai Institutes for Biological Sciences, Chinese Academy of Sciences, 200031 Shanghai, China; 10 Children’s Hospital of Fudan University, 399 Wanyuan Road, 201102 Shanghai, China; 11 Institutes of Biomedical Sciences, Fudan University, 200032 Shanghai, China

**Keywords:** Stem-cell research, Development

## Abstract

Duplication of *MECP2* (Methyl-CpG-binding protein 2) causes severe mental illness called *MECP2* duplication syndrome (MDS), yet the underlying mechanism remains elusive. Here we show, in *Tg(MECP2)* transgenic mouse brain or cultured neural progenitor cells (NPCs), that elevated MeCP2 expression promotes NPC differentiation into neurons. Ectopic expression of MeCP2 inhibits ADAM10 and thus the NOTCH pathway during NPC differentiation. In human cells, this downregulation on ADAM10 was mediated by miRNA-197, which is upregulated by MeCP2. Surprisingly, miR-197 binds to the ADAM10 3′-UTR via its 3′ side, not the canonical seed sequence on the 5′ side. In mouse cells, a noncoding RNA Gm28836 is used to replace the function of miR-197 between MeCP2 and ADAM10. Similar to MeCP2, overexpressing miR-197 also promotes NPCs differentiation into neurons. Interestingly, three rare missense mutations (H371R, E394K, and G428S) in *MECP2*, which we identified in a Han Chinese autism spectrum disorders (ASD) cohort showed loss-of-function effects in NPC differentiation assay. These mutations cannot upregulate miR-197. Overexpressing miR-197 together with these MeCP2 mutations could rescue the downregulation on ADAM10. Not only the inhibitor of miR-197 could reverse the effect of overexpressed MeCP2 on NPCs differentiation, but also overexpression of miR-197 could reverse the NPCs differentiation defects caused by *MECP2* mutations. Our results revealed that a regulatory axis involving MeCP2, miR-197, ADAM10, and NOTCH signaling is critical for NPC differentiation, which is affected by both MeCP2 duplication and mutation.

## Introduction

*MECP2* (methyl-CpG binding protein 2), an X-linked gene encoding the methyl-cytosine binding protein MeCP2, is associated with two severe neurological disorders, Rett syndrome (RTT) and *MECP2* duplication syndrome (MDS), which result from loss and gain of function of *MECP2*, respectively. Although clinically distinguished from ASD by the Diagnostic and Statistical Manual of Mental Disorders (DSM-5) [1], autistic features are often observed (>60%) in RTT patients [2, 3]. Meanwhile, ~100% MDS patients have autistic-like behaviors [4, 5]. Therefore, both functional and dosage variations of MeCP2 are tightly associated with brain development and functions [3, 6]. MeCP2 was originally identified as a transcriptional repressor [7, 8], but recent studies have shown it has diverse functions, including transcription activation [9], mRNA splicing [10, 11], and microRNA (miRNA) processing [12, 13], to name but a few. As both RTT and ASD patients show symptoms shortly after birth, most functional studies of MeCP2 have been focused on postnatal events such as dendritic arborization [14–16], synapse formation and plasticity [16–18], and adult neurogenesis [19–21]. However, the function of MeCP2 during embryonic CNS development is still elusive.

MeCP2 is widely and highly expressed in the developing central nervous system (CNS), including both the early neural tube and embryonic forebrain, in zebrafish, chicken, and mouse [22–25]. However, embryonic development seems to be unaffected in *Mecp2* knockout mice [26, 27]. But studies in *Xenopus* showed that MeCP2 promoted neurogenesis of *Xenopus* embryos, while the RTT mutant R168X failed to do so [28]. A recent study in the monkey showed that TALEN-edited mutation of *MECP2* caused embryonic lethality in male mutant monkeys [29]. Furthermore, human iPSCs generated from RTT patients with dysfunctional MeCP2 have abnormal neurogenesis and gliogenesis [30, 31]. Therefore, it is likely that MeCP2 has an important role during early CNS development.

In the present study, we demonstrated that overexpressed MeCP2 promoted neurogenesis in both primary cultured NPCs and embryonic brain in the MDS model Tg(*MECP2*) mouse. Our results revealed a novel mechanism involving miR-197, ADAM10 (A disintergrin and metalloprotease 10), and NOTCH signaling as a critical regulatory axis for the enhanced neurogenesis induced by MeCP2. Furthermore, we identified three rare missense *MECP2* mutations (H371R, E394K, and G428S) in ASD patients in our Han Chinese ASD cohort, which are novel in the East Asian population according to ExAC [32]. These *MECP2* mutations resulted in dysfunctional regulation of miR-197 and neurogenesis. Not only the inhibitor of miR-197 could reverse the effect of overexpressed MeCP2, but also overexpression of miR-197 could reverse the NPCs differentiation defects caused by *MECP2* mutations. Our results revealed a novel regulatory pathway via miR-197 by which MeCP2 acting on ADAM10/NOTCH signaling, implicating that molecules in this pathway are important for the etiology of MDS and possibly for ASD.

## Results

### Neurogenesis is enhanced in Tg(*MECP2*) mouse fetal brain and cultured NPCs

**Fig. 1:**
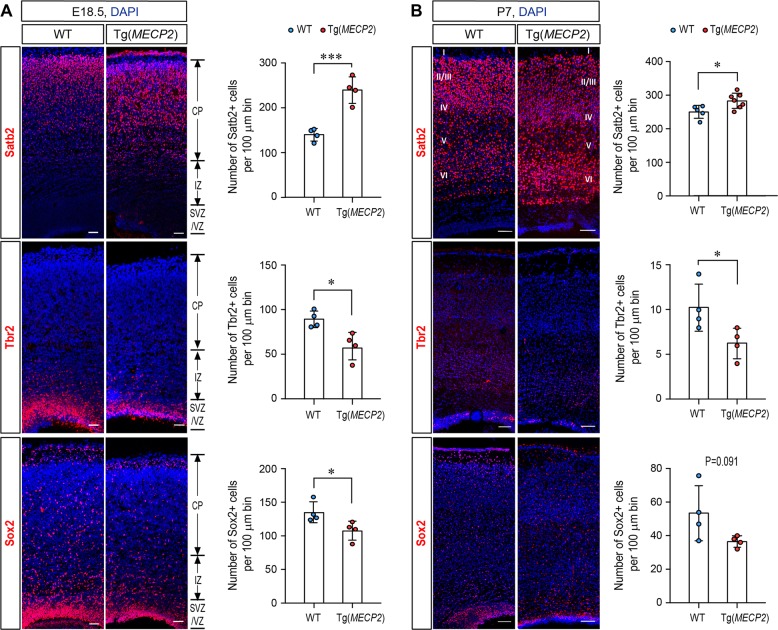
There are more Satb2 positive neurons in Tg*(MECP2)* FVB mice cortex. Coronal brain sections from E18.5 (**a**) and P7 (**b**) Tg(*MECP2*) FVB mice or WT littermates were stained with cortical neuron marker Satb2, and progenitor markers Sox2 and Tbr2. *N* ≥ 4. DAPI (blue) was used for nuclear staining. The numbers of positive cells within 100μm bin were counted. Representative sections are shown in left and statistical analysis are shown in right panels, respectively. All statistic data represent means ± SEM. *p < 0.05, ***p < 0.001. Scale bar is 50 μm in E18.5 and 100μm in P7

To determine the effect of *MECP2* duplication on cell fate in vivo in fetal brain, we investigated a Tg(*MECP2*) mouse line which had previously been used as a model for MDS, as it contains an extra copy of human *MECP2* and exhibits approximately doubled MeCP2 levels in the brain [33]. For quantification of cell fate in, Immunofluorescent staining on E18.5 and P7 brain sections from the wild-type (WT) and Tg(*MECP2*) mice revealed that there are significantly more Satb2^+^ cortical neurons in CP layer and significantly less Sox2^+^ and Tbr2^+^ progenitor cells in VZ/SVZ layer of E18.5 Tg*(MECP2)* mice compared to WT littermates (Fig. 1a). By P7, there are still significantly more Satb2^+^ neurons present in P7 Tg*(MECP2)* mice cortex than WT cortex (Fig. 1b).

**Fig. 2:**
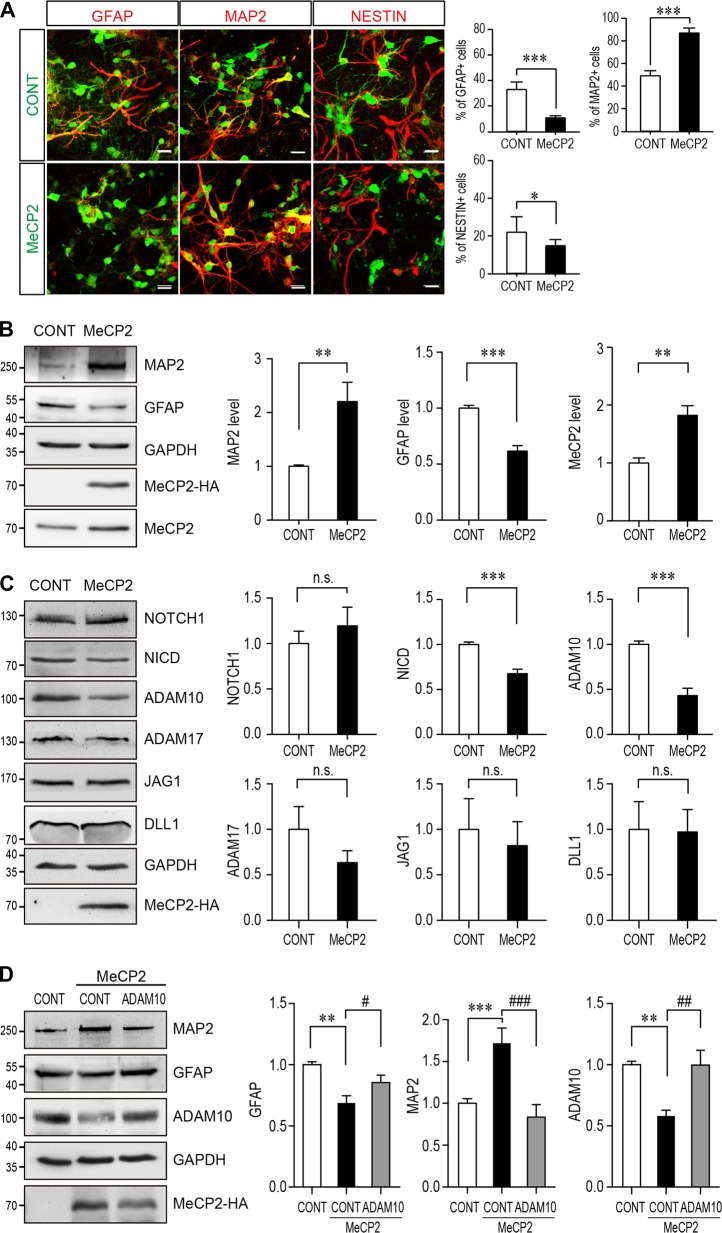
*MECP2* duplication promotes neurogenesis of cultured NPCs and ADAM10 is a critical down-stream molecule of MeCP2 in regulating NPCs differentiation. Mouse primary NPCs isolated from C57BL/6 mouse E12.5 embryonic cortex were infected with MeCP2 expressing lentivirus and cultured for 72 h. Cells were subjected for immunofluorescent staining (**a**) and cell lysates were subjected to western blotting (**b**–**d**). **a** Immunofluorescent staining for GFAP, MAP2, and NESTIN were performed on NPCs (*N* = 9). The MeCP2 and control viruses contain EGFP and showed here as green. The staining for MAP2, GFAP, and NESTIN are labeled with pseudo-colored red. Representative images are shown on the left and the percentage of Nestin^+^, MAP2^+^ and GFAP^+^ cells within EGFP^+^ cells are shown on the right panels respectively. Scale bar is 50 μm. **b** Western blot analysis for MAP2, GFAP, HA labeled MeCP2 and total MeCP2 (*N* = 9). GAPDH was used as loading control. Representative blot is shown on the left, and statistical analysis for MAP2, GFAP and MeCP2 levels are shown in the right panels, respectively. **c** Western blot analysis for components of NOTCH pathway, including NOTCH1, NICD, ADAM10, ADAM17, JAG1, and DLL1 (*N* ≥ 5). GAPDH was used as a loading control. Representative blots are shown on the left and statistical analyses for each component were presented on the right panels. **d** Overexpression of ADAM10 together with WT MeCP2 could reverse the differentiation effects of MeCP2 in cultured NPCs. HA tagged MeCP2 were transfected with either control vector or ADAM10 expressing plasmid (*N* ≥ 5). Seventy-two hours later, NPCs lysates were subjected to Western blot analysis for MAP2, GFAP, ADAM10, and HA. GAPDH was used as a loading control. Representative blots are shown on the left panel and statistical analyses were presented on the right panel, respectively

Primary NPCs were isolated from E12.5-14.5 mouse cortex to further examine the in vitro effect of MeCP2 overexpression on NPCs differentiation. WT NPCs from C57BL/6 mice were infected with MeCP2 lentivirus after seeding on the culture dish. The exogenous expression could also double the expression levels of MeCP2 in those NPCs (Fig. 2b). Both transiently overexpressed MeCP2 in C57BL/6 NPCs and elevated MeCP2 in NPCs isolated from transgenic Tg(*MECP2*) mice significantly upregulated the level of the neuronal marker MAP2, and downregulated the level of the glia marker GFAP in NPCs (Fig. 2b, Fig. S1). Similarly, immunofluorescent staining on cultured WT NPCs infected by lentivirus also showed significantly more MAP2^+^, less GFAP^+^ and less Nestin^+^ cells with MeCP2 overexpression (Fig. 2a). Taken together, the results demonstrate that elevated MeCP2 expression affects NPCs cell fate and promotes neurogenesis.

### ADAM10 is downstream of MeCP2 in NPCs differentiation

Since NOTCH is a key molecule for NSCs fate and differentiation [34, 35], we next examined the levels of several key molecules in the NOTCH pathway in primary cultured NPCs after overexpressing MeCP2. The levels of two NOTCH ligands JAG1 and DLL1, the full-length receptor NOTCH1, the activated NOTCH intracellular domain (NICD), and the two rate-limiting S2 enzymes for NOTCH cleavage, ADAM10 and ADAM17, were examined by Western blot analysis in primary NPCs transfected with either MeCP2 or an empty control vector. The results showed that the levels of NICD and ADAM10 were significantly downregulated by MeCP2 expression in NPCs (Fig. 2c), while ADAM17, full-length NOTCH1, JAG1, and DLL1 were not significantly affected (Fig. 2c). When ADAM10 was expressed together with MeCP2 in primary cultured NPCs, it could reverse the effect of MeCP2 overexpression as indicated by the restoration of MAP2 and GFAP levels close to those of controls (Fig. 2d). These results suggest that ADAM10 is functionally downstream of MeCP2 during neurogenesis.

### MeCP2 downregulates human ADAM10 expression through upregulating miR-197

**Fig. 3:**
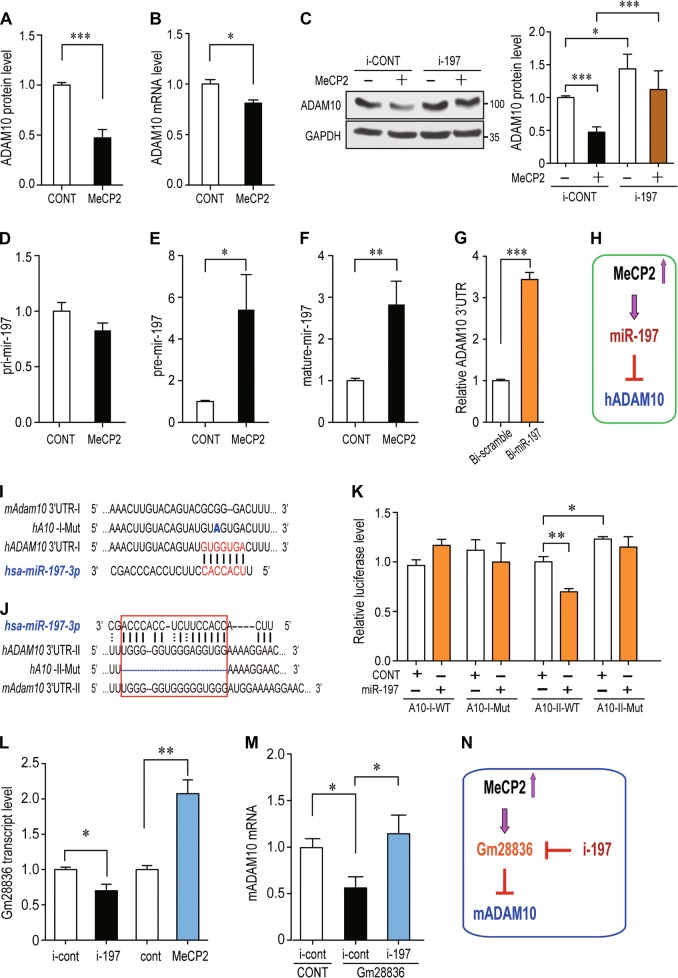
MiR-197 is upregulated by MeCP2 to downregulate human ADAM10 while ncRNA Gm28836 is upregulated by MeCP2 to downregulate mouse ADAM10. **a**, **b** Human glioblastoma cells U251 were transfected with empty control or plasmid to overexpress MeCP2 (*N* ≥ 5), and subjected to either western blotting or qRT-PCR to detect ADAM10 protein (**a**) or mRNA (**b**). **c** U251 cells were transfected with MeCP2 and a miR-197 inhibitor (i-197) or scramble control, and subjected to Western blotting for ADAM10 protein (*N* ≥ 3). GAPDH was used as loading control in both experiments. **d**–**f** U251 cells were transfected with either empty vector or MeCP2 expressing plasmids and cultured for 24 h (*N* = 9). RNA from these cells were extracted and subjected for qRT-PCR. The levels of pri-miR-197, pre-miR-197, and mature miR-197 were shown in **d**–**f**, respectively. **g** Biotin-labeled miR-197 and control scramble were transfected into U251 cells and the human *ADAM10* 3′-UTR pulled down by miR-197 or control scramble were quantified by qRT-PCR (*N* = 4). **h** A scheme illustration for MeCP2 regulation on miR-197 and human ADAM10. **i** A 7mer-m8 miR-197 binding site predicted by TargetscanHuman. The alignment of miR-197 to human and mouse *ADAM10* 3′-UTR and the point mutation G>A in A10-I-Mut are illustrated. **j** A miR-197 binding site predicted by RNAhybrid is highly conserved between human and mouse. The deletion mutation in A10-II-Mut was also illustrated. **k** Luciferase reporter plasmids for different regions of *ADAM10* 3′-UTR were transfected into U251 cells with miR-197 mimics or control (*N* ≥ 8). miR-197 only downregulates the A10-II-WT which contains the region illustrated in **j**. **l** Mouse neuroblastoma cell line Neuro-2a (N2a) cells were transfected with negative control or i-197, empty vector or MeCP2 expressing plasmid. Twenty-four hours later, total RNA was extracted and subjected to qRT-PCR to determine the level of Gm28836 RNA. Mouse Gapdh was used as internal control. (*N* = 3) **m** Mouse N2a cells were transfected empty vector, Gm28836 plus i-197 or its negative control. The ratio of i-197 to Gm28836 was 1:1. Twenty-four hours later, total RNA was extracted and subjected to qRT-PCR to determine the level of mouse Adam10. Mouse Gapdh was used as internal control. (*N* = 4) **n** A scheme illustration for MeCP2 regulation on mouse ADAM10 via Gm28836. All statistical data are represented as means ± SEM. *p < 0.05, **p < 0.01, ***p < 0.001

We next investigated how MeCP2 regulates ADAM10. A human glioblastoma cell line U251 was used to overexpressing MeCP2. Similar to the cultured mouse NPCs, the ADAM10 protein level was also significantly down-regulated (~53%) by MeCP2 overexpression in U251 cells (Fig. 3a). We noticed that the mRNA level of ADAM10 was downregulated ~19% by MeCP2 (Fig. 3b). Since ADAM10 was not identified as a transcriptional target of MeCP2 [9], we screened the potential microRNAs (miRNAs) that might be involved in ADAM10 regulation by MeCP2. Seven miRNAs, identified as the most significantly regulated miRNAs by MeCP2 in the adult NSCs of the *Mecp2*-/y mouse [36], were used for our studies. When inhibitors for each miRNA (e.g., i-197 as an inhibitor of miR-197) were transfected into U251 cells, ADAM10 protein levels were significantly upregulated by i-222, i-193, and i-197; while the other four inhibitors had no statistically significant effect (Fig. S2A). However, when MeCP2 was co-transfected with miRNA inhibitors, only i-197 and i-193 were able to reverse the MeCP2-induced downregulation of ADAM10 protein (Fig. 3c and Fig. S2B). The expression profile from miRNAMap shows that hsa-miR-193 is primarily expressed in the muscle, and hsa-miR-197 is highly expressed in the brain (http://mirnamap.mbc.nctu.edu.tw) [37]; therefore, miR-197 is the most likely target of MeCP2 in the regulation of ADAM10 expression in the brain.

We subsequently investigated how MeCP2 regulates miR-197. RT-PCR experiments showed that the levels of pre- and matured miR-197 were significantly upregulated by approximately 5.4 and 2.7-fold by MeCP2, respectively (Fig. 3e, f), while the level of pri-miR-197 was not affected by MeCP2 (Fig. 3d). In addition, RNA-IP assay with a biotinylated miR-197 demonstrated that human *ADAM10* 3′-UTR could be pulled-down by miR-197 (Fig. 3g). Taken together, MeCP2 upregulates miR-197, which binds to the 3′-UTR of ADAM10 and downregulates ADAM10 (Fig. 3h).

We went on to investigate the potential target site on *ADAM10* 3′-UTR for miR-197. A 7mer-m8 miR-197 binding site at position 1568-1574 of human *ADAM10* 3′-UTR was predicted by TargetscanHuman (http://www.targetscan.org/vert_71/) [38], which is poorly conserved between human and mouse (position 1563–1568 of mouse *Adam10* 3′-UTR) (Fig. 3i). A luciferase reporter A10-I-WT was constructed with a 305 bp fragment of human *ADAM10* 3′-UTR covering this predicted position 1568–1574. The G to A point mutation in the seed sequence was also constructed as A10-I-Mut (Fig. 3i). Both constructs were transfected into U251 cells with either miR-197 mimics or mimic control. Surprisingly, the results showed that miR-197 had no effect on either A10-I-WT nor A10-I-Mut (Fig. 3k), rather the point mutation blocked the interaction of human *ADAM10* 3′-UTR with miR-224, which has an overlapped seed sequence at position 1571-1577 (Fig. S3A, B).

Further examination by RNAhybrid analysis [39], a different miR-197 binding site at position 186-201 of human *ADAM10* 3′-UTR was predicted based on the free energy of the miRNA-target-duplex, which is highly conserved between human and mouse (position 185-199 of mouse *Adam10* 3′-UTR) (Fig. 3j). This binding site is different from traditional miRNAs and it was predicted to bind the 3′ side of miR-197. A luciferase reporter A10-II-WT was constructed with a 402 bp fragment of human *ADAM10* 3′-UTR covering positions 186-201. A deletion mutant reporter A10-II-Mut, deleting this untraditional miRNA binding site, was also constructed (Fig. 3j). MiR-197 mimics significantly downregulated the relative luciferase level of A10-II-WT but not A10-II-Mut (Fig. 3k). These results suggested that miR-197 interacts with ADAM10 3′-UTR in a non-canonical way.

### MeCP2 downregulates mouse ADAM10 expression through a ncRNA Gm28836

The mouse miR-197 was removed from miRBase in 2014 based on the fact that “the sequence does not map in a stem-loop region of the genomic sequence or any known mouse transcript sequence”; however, we noticed that small RNA libraries were used to identify miR-197 in both human and mouse in the original paper by Landgraf et al. [40]. Indeed, a small RNA could be PCR amplified from E18.5 mouse brain by using has-miR-197 specific primers (Fig. S3C). Sequencing results revealed the PCR product from mouse E18.5 brain has 16nt identical to the 3′ side of human miR-197 (Fig. S3D). This 16nt sequence was used to blast the mouse genomic plus transcript (mouse G+T) in the NCBI database (https://blast.ncbi.nlm.nih.gov). The results showed that there are three loci in the mouse genome have identical 16nt sequences and several predicted noncoding RNAs (ncRNAs) have 15 identical nucleotides, including Gm16196, Gm41705, and Gm28836 (Table S1). We designed primers to detect whether these ncRNAs are expressed in the mouse E18.5 brain. Our results showed that Gm28836 is expressed in the mouse E18.5 brain (Figure S3E) and the expression levels of Gm28836 is upregulated by MeCP2 and down-regulated by i-197, the miR-197 inhibitor (Fig. 3l). What’s more, Gm28836 can down-regulate mouse ADAM10 and this effect can be blocked by i-197 (Fig. 3m). Therefore, even though there is no conserved miR-197 in the mouse brain, the molecular pathway is still conserved in mouse by using Gm28836 to replace the function of miR-197 between MeCP2 and ADAM10 (Fig. 3n).

### MiR-197 mimics the function of MeCP2 in promoting mouse NPCs differentiation

**Fig. 4:**
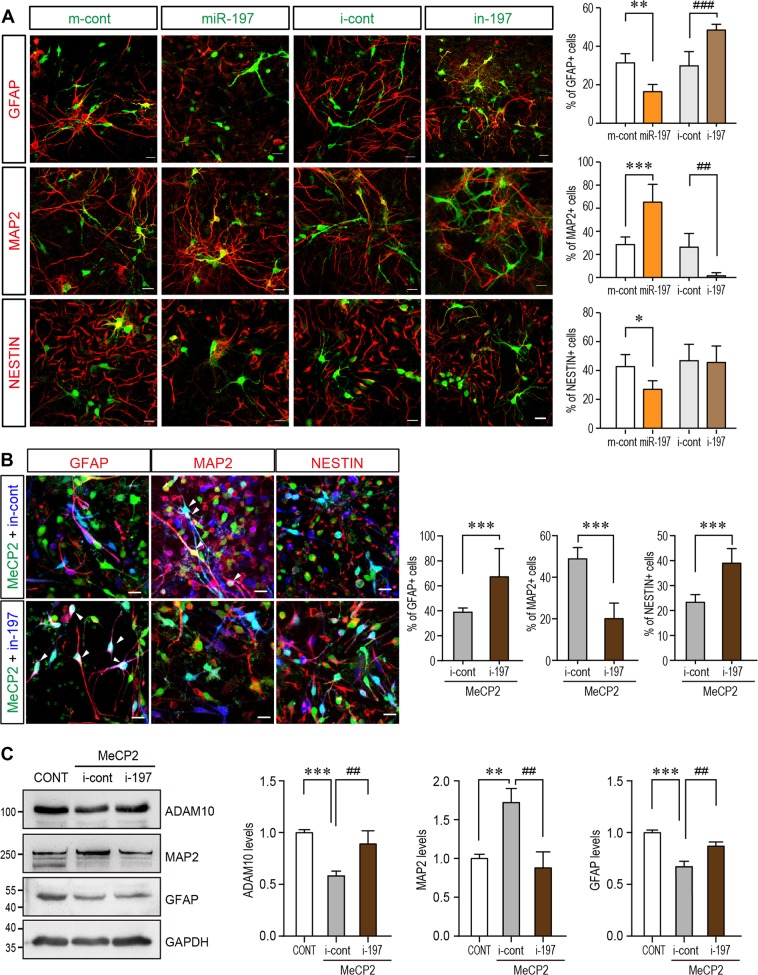
miR-197 promotes neurogenesis and the inhibitor of miR-197 reverses the effect of MeCP2 overexpression. **a** Primary NPCs from C57BL/6 mouse were infected with assorted lentivirus to over-express either miR-197, miR-control, miR-197 inhibitor (i-197), or inhibitor-control (*N* ≥ 4). Seventy-two hours later, cells were subjected for immunofluorescent staining. The infected cells are labeled with pseudo-colored green. The staining for MAP2, GFAP, and NESTIN are labeled with pseudo-colored red. The percentage of Nestin^+^, MAP2^+^, and GFAP^+^ cells within infected cells are shown on the right side respectively. **b** Primary mouse NPCs were infected with assorted lentivirus to overexpress MeCP2 with either i-197 or i-cont (*N* = 9). Seventy-two hours later, cells were subjected for immunofluorescent staining for MAP2, GFAP, and NESTIN. The MeCP2 and control viruses contain EGFP and showed here as green. The i-197/i-cont viruses infected cells are pseudo-color labeled as blue. The staining for MAP2, GFAP, and NESTIN are labeled with pseudo-colored red. The percentage of Nestin^+^, MAP2^+^, and GFAP^+^ cells within green and blue double positive cells (cyan in nucleus, white arrow head) are shown on the right side, respectively. **c** Primary NPCs from C57BL/6 mouse were transfected with MeCP2 expressing plasmid together with either inhibitor control or i-197 (*N* ≥ 4). Cell lysates were subjected to Western blot analysis for MAP2, GFAP, and ADAM10. Representative blots are shown on the left side and statistical analyses are shown on the right side, respectively. All statistical data are presented as means ± SEM. * or #*p* < 0.05, ** or ## p < 0.01, *** or ### *p* < 0.001. Scale bar is 50 μm

Since the molecular pathway is conserved in mouse, we overexpressed miR-197 in primary cultured NPCs to observe its effect on NPCs differentiation. Immunofluorescent staining showed that miR-197 affects NPCs fate and differentiation similar to MeCP2, as evidenced by an elevated ratio of MAP2^+^ and decreased ratio of GFAP^+^ and Nestin^+^ cells in the differentiated NPCs (Fig. 4a). The inhibitor of miR-197 (i-197) had opposite effect with decreased ratio of MAP2^+^ and elevated ratio of GFAP^+^ cells (Fig. 4a). Furthermore, when i-197 was co-expressed with MeCP2 in NPCs, it could reverse the effect of MeCP2 (Fig. 4b). MeCP2 induced down-regulation of ADAM10 was also blocked by i-197 (Fig. 4c). These data showed that ectopic expression of miR-197 mimics the function of MeCP2 during mouse NPCs differentiation.

### *3MECP2* mutations identified in a Chinese ASD cohort failed to upregulate miR-197

A targeted-sequencing of *MECP2* gene exons was performed in a Han Chinese cohort consisting of 288 ASD patients and 369 controls. Five rare missense *MECP2* mutations were identified in six male ASD patients that were not observed among any of our controls (Table 1). These mutations were c.590C>T, c.695G>C, c.1112A>G, c.1180G>A, and c.1282G>A, which correlated to T197M, G232A, H371R, E394K, and G428S in the human MeCP2-e1 protein (mouse MeCP2-e2) (Fig. S4), represents the isoform highly expressed in the brain [24, 41]. The patients carrying these mutations showed a spectrum of symptoms, and the patient carrying the G428S mutation presented the most severe symptoms including severe intellectual disability with no functional language skills, and other medical conditions such as sleep disturbances (Table 1). According to ExAC, three C-terminal mutations (H371R, E394K, and G428S) were not previously identified in the East Asian population (Total 4327 samples, with Male/Female = 2016/2311) [32]. The recurrent (two patients) mutation H371R in our cohort represents a novel mutation in all ExAC populations, a database which currently contains sixty thousand individuals [32]. Therefore, we focused on those C-terminal mutations in the follow-up studies.

**Table 1:** Rare mutations of *MECP2* detected in Chinese ASD cohort

cDNA	Amino acid	Domain location	Patient	Birth Year	Sex	Intellectual disability	Functional language	Other medical issues	Mother	AF in ASD^a^	AF in Cont^b^	AF in ExAC	*p* value^d^	AF in ExAC EA^C^
590C>T	T197M	Interdomain	ASD164	2007	M	NO	Yes	NO	Carrier	0.00303 (1/330)	0	0.0005475 (48/87676)	0.169	0.0004519 (3/6638)
695G>C	G232A	TRD	ASD97	2002	M	NO	Yes	ADHD	Carrier	0.00303 (1/330)	0	0.0001942 (17/87526)	0.066	0.00196 (13/6634)
1112A>G	H371R	C-term	ASD348 ASD349	2008 2007	M M	Yes (mild) NO	NO Yes	ADHD ADHD	Carrier Carrier	0.00606 (2/330)	0	0	1.4e−05	0
1180G>A	E394K	C-term	ASD243	2004	M	Yes (moderate)	NO	ADHD/mood disorder	NA	0.00303 (1/330)	0	3.627e-05 (3/82722)	0.016	0 (0/6429)
1282G>A	G428S	C-term	ASD241	2006	M	Yes (Severe)	NO	ADHD/mood disorder /Sleep disturbance	Carrier	0.00303 (1/330)	0	0.0001256 (11/87586)	0.044	0 (0/6634)

*AF* allele frequency, *EA* East Asian, *M* male, *NA* not available

^a^AF in our ASD cohort. All five mutations are case specific. Our Chinese ASD cohort includes 288 Chinese ASD cases with Male/Female = 246:42. Therefore, the adjusted allele number is 330

^b^AF in our 369 controls with Male/Female = 300:69. The adjusted allele number is 438

^c^AF in East Asian population in ExAC database with Male/Female = 2016/2311. The adjusted allele number is 6638

^d^*p* value is calculated between AFs in Chinese ASD and ExAC by Fisher’s exact test

**Fig. 5:**
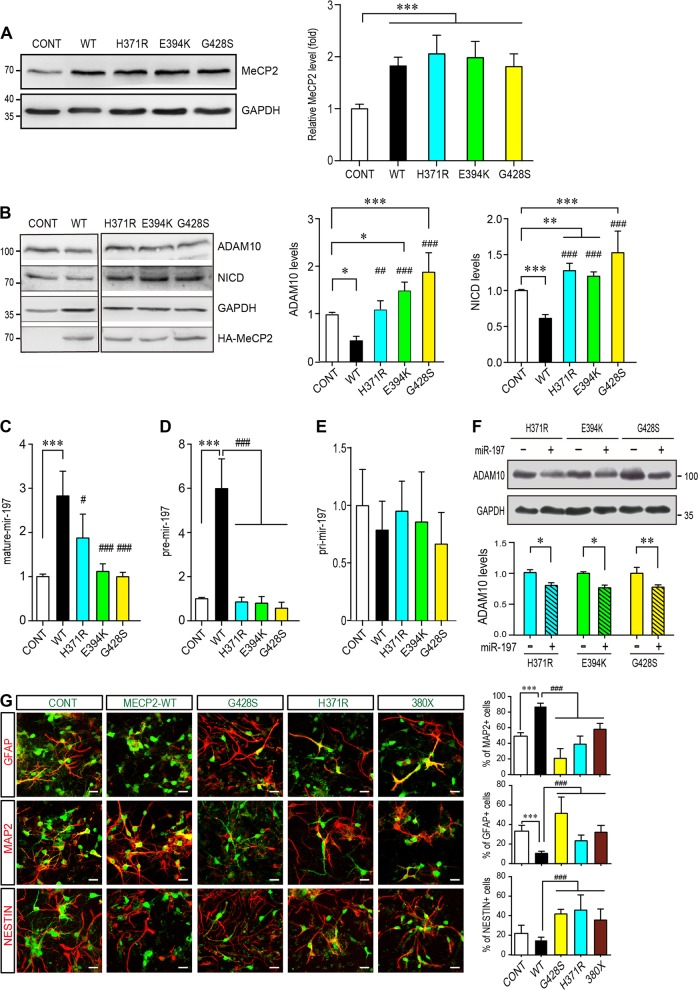
ASD-related MeCP2 mutants lose the effect on promoting miR-197. **a** Mouse primary NPCs isolated from C57BL/6 mouse E12.5 embryonic cortex were transfected with either control vector or different MeCP2 expressing plasmids and cultured for 72 h. Cell lysates were subjected to western blot analysis for MeCP2 protein level and GAPDH was used as a loading control. Representative blot is shown on the left, and statistical analysis for MeCP2 levels are shown on the right side. *N* = 3. **b** Primary NPCs were transfected with either WT or three C-terminal MeCP2 mutant expressing plasmids and cultured for 72 h. Cell lysates were subjected to western blot analysis for ADAM10, NICD, and HA. Representative blots are presented on the left side and statistical analyses for cells in each layer are shown on the right side. GAPDH was used as internal control (*N* ≥ 4). All data represent means ± SEM. Effects of the mutations are either compared to control (indicated with *), or WT MeCP2 (indicated with #). **c**–**f** U251 cells were transfected with either WT or mutant MeCP2 expressing plasmids and cultured for 24 h (*N* ≥ 4). RNA from these cells was subjected to qPCR for either mature miR-197 (**c**), pre-miR-197 (**d**), or pri-miR-197 (**e**). **f** U251 cells were transfected with mutant MeCP2 expressing plasmids and either control or miR-197 plasmid (*N* ≥ 5). Cells were cultured for 24 h and cell lysates were subjected to western blot for ADAM10. Representative blots are shown on the left side and statistical analyses are shown on the right side, respectively. **g** Primary mouse NPCs were infected with assorted lentivirus to overexpress WT MeCP2, MeCP2^H371R^, MeCP2^G428S^, and MeCP2^380×^ (*N* = 9). Seventy-two hours later, cells were subjected for immunofluorescent staining for MAP2, GFAP, and NESTIN. The infected cells are labeled with EGFP (green). The staining for MAP2, GFAP, and NESTIN are labeled with pseudo-colored red. The percentage of MAP2^+^, GFAP^+^, and Nestin^+^ cells within EGFP^+^ cells are shown on the right, respectively. All statistical data are presented as means ± SEM. * or # *p* < 0.05, ** or ## *p* < 0.01, *** or ### *p* < 0.001. Scale bar is 50 μm

When overexpressed, these mutations did not affect the expression levels of MeCP2 in NPCs (Fig. 5a). But these three MeCP2 C-terminal mutants lost the ability to downregulate ADAM10 and NICD expression in primary culture mouse NPCs (Fig. 5b). Further examination by qRT-RNA experiments also showed that these three MeCP2 C-terminal mutants failed to upregulate pre-miR-197 (Fig. 5d) and matured miR-197 (Fig. 5c) without affecting pri-miR-197 (Fig. 5e) in U251 cells. When miR-197 was co-expressed with three MeCP2 C-terminal mutants, the downregulation on ADAM10 was restored in presence of the MeCP2 mutants (Fig. 5f).

### The ASD-related MeCP2 mutants are loss-of-function mutants in NPCs differentiation

We next examined the effects of the ASD-related MeCP2 mutants in NPCs differentiation assay. Since H371R is the recurrent mutation and the patient carrying G428S mutation showed most severe symptoms, we focused on these two mutants in NPCs differentiation assay. When overexpressed in NPCs, MeCP2^H371R^ showed loss-of-function effects similar to that of MeCP2^380×^, a previously reported loss-of-function truncation mutant [12], as they resulted in less percentage of MAP2^+^ cells (Fig. 5g).

**Fig. 6:**
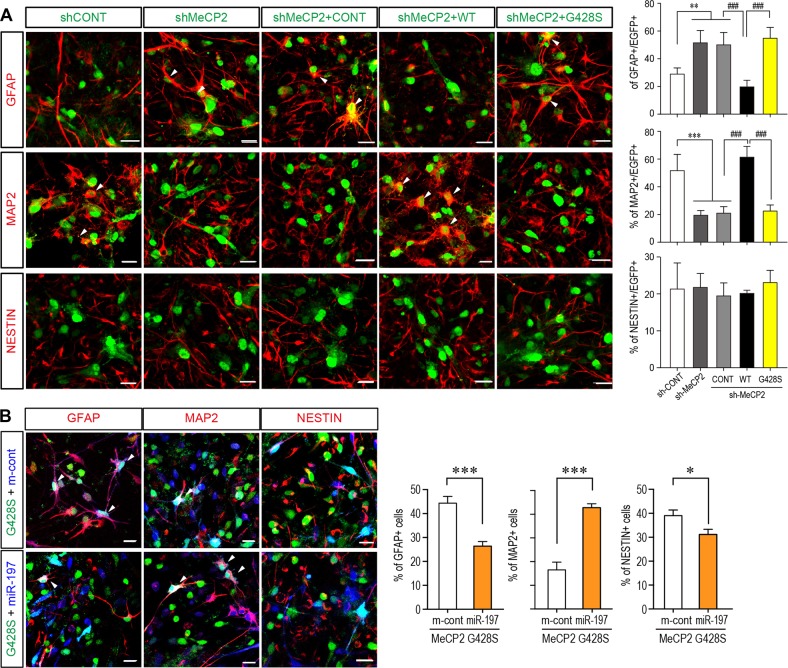
MeCP2-G428S is a loss-of-function mutant in NPC differentiation and miR-197 could reverse the defects caused by MeCP2-G428S in NPCs. **a** Primary mouse NPCs were infected with sh-MeCP2 lentivirus to knock-down endogenous mouse MeCP2. Sixteen hours later, these cells were infected again with control vector, WT MeCP2, or MeCP2-G428 virus (*N* = 4). Seventy-two hours later, cells were subjected for immunofluorescent staining. The infected cells are labeled with pseudo-colored green. The staining for MAP2, GFAP, and NESTIN are labeled with pseudo-colored red. The percentage of Nestin^+^, MAP2^+^, and GFAP^+^ cells within infected cells are shown on the right side respectively. **b** Primary mouse NPCs were infected with MeCP2-G428S lentivirus with either miR-control (m-cont) or miR-197 virus (*N* = 5). Seventy-two hours after infection, cells were subjected for immunofluorescent staining. The viruses contain EGFP and showed here as green. The miR-197/m-cont viruses infected cells are pseudo-color labeled as blue. The staining for MAP2, GFAP, and NESTIN are labeled with pseudo-colored red. The percentage of Nestin^+^, MAP2^+^, and GFAP^+^ cells within green and blue double positive cells (cyan in nucleus, white arrow head) are shown on the right side, respectively. All statistical data are presented as means ± SEM. ## *p* < 0.01, *** or ### *p* < 0.001. Scale bar is 50 μm

We noticed that overexpression of the MeCP2^G428S^ mutant in NPCs further reduced neuronal differentiation as indicated by significantly reduced percentage of MAP2^+^ cells (~21%) compared to either WT MeCP2 (~87% MAP2^+^) or the control empty vectors (~49% MAP2^+^) (Fig. 5g). However, when the endogenous MeCP2 was blocked by shRNA, MeCP2^G428S^ showed only loss-of-function effect in promoting neurogenesis as overexpressing MeCP2^G428S^ did not change the percentage of GFAP^+^ cells as compared to sh-MeCP2 knock-down alone (Fig. 6a). And this loss-of-function on NPCs differentiation can be restored by overexpressing miR-197 together with MeCP2^G428S^ in NPCs (Fig. 6b).

### Regulating miR-197 reversed the neurogenesis effects caused by both MeCP2 duplication and mutant in electroporated cortex

**Fig. 7:**
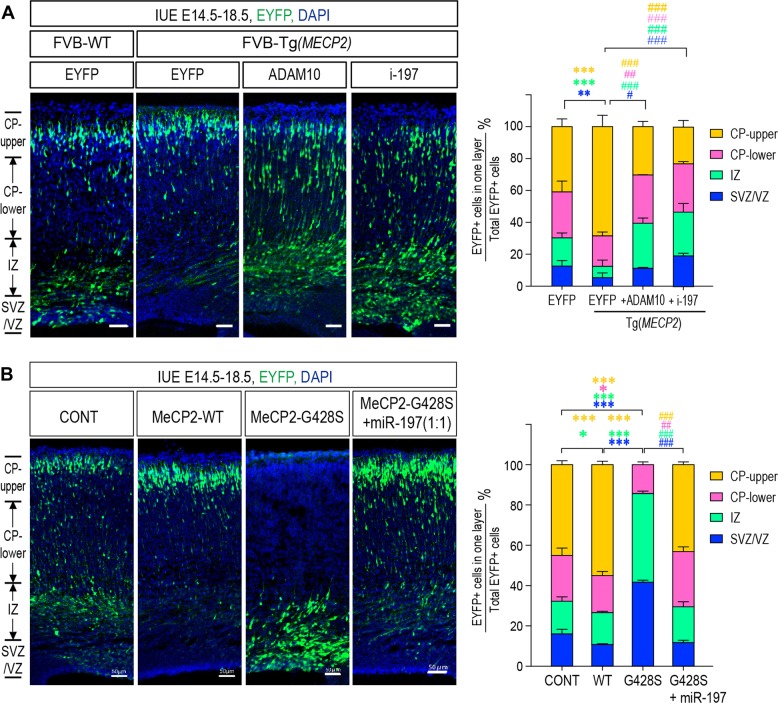
Regulating miR-197 reverses the effects of MeCP2 duplication and MeCP2-G428S. **a** Fetal *Tg(MECP2)* or WT FVB mice cortex were electroporated with EYFP alone or EYFP with either ADAM10 or i-197 at E14.5. All samples were collected at E18.5 for sectioning and immunostaining. Each condition was repeated in four different embryos from four pregnant female mice (*N* = 4). **b** C57BL/6 mouse were electroporated at E14.5 with either control EYFP alone or EYFP with MeCP2, MeCP2-G428S, or MeCP2-428S + miR-197 (1:1 ratio) (*N* = 4). Again, samples were collected at E18.5 for sectioning and immunostaining. Representative brain sections are presented on the left and the EYFP^+^ cells in each layer were counted and compared to the total EYFP^+^ cells. The statistical analyses for cells in each layer are presented on the right panel. DAPI (blue) was used for nuclear staining. Scale bar is 50 μm. All statistic data represent means ± SEM. *, # *p* < 0.05, **, ## *p* < 0.01, ***, ### *p* < 0.001

To test the in vivo effects of regulating miR-197, we applied in utero electroporation (IUE) on mouse embryonic cortex. During cortical neurogenesis in the mouse, NSCs/NPCs in the ventricular (VZ) and subventricular (SVZ) zones are differentiated into immature neurons and migrate radially to the cortical plate (CP) to become mature neurons [42, 43]. Using IUE, EYFP expressing construct were electroporated into embryonic cortex at E14.5 to label one group of NSC/NPCs. Thus, the differentiation/migration of this group of labeled NSC/NPCs could be inspected at E18.5 using immunofluorescent staining. As shown in Fig. 7a, there were significantly less EYFP^+^ cells in the VZ/SVZ progenitor layer and significantly more EYFP^+^ cells that had reached the upper CP layer in the E18.5 Tg*(MECP2)* cortex, compared to those in control WT FVB mice. This effect was also observed in C57BL/6 WT cortex ectopically overexpressing MeCP2 (Fig. 7b). On the other hand, most of the MeCP2^G428S^ overexpressing cells remaining within the VZ/SVZ layers in mouse cerebral cortex, and very few cells reached the CP at E18.5 (Fig. 7b). Interestingly, expression of ADAM10 and i-197 in Tg(*MECP2*) mice could reverse the effect of elevated MeCP2 expression, as significantly more EYFP^+^ cells remained in the VZ/SVZ and IZ layers and fewer EYFP^+^ cells reached the upper CP layer (Fig. 7a); while exogenous miR-197 could almost completely reverse the effect of MeCP2^G428S^ in WT mouse cortex (Fig. 7b).

## Discussion

In this study, we demonstrated that the embryonic NSC/NPC differentiation is tightly regulated by the dosage and function of MeCP2. In a MDS animal model that elevated MeCP2 expression promotes neurogenesis. We discovered that MeCP2 upregulates miR-197 to downregulate ADAM10 during neurogenesis. The function of miR-197 is replaced by a ncRNA Gm28836 in mouse. Moreover, we identified three rare missense mutations in *MECP2* in an ASD cohort and found that they are loss-of-function mutants in regulating NPC differentiation. Interestingly, miR-197 can reverse the differentiation defects caused by the ASD-related MeCP2 mutants. Our results revealed that miR-197 plays critical role in MeCP2 regulated NPCs differentiation via ADAM10/NOTCH pathway.

MiR-197 is an interesting miRNA as homology genes were only identified in five primates (human, rhesus monkey, western gorilla, chimpanzee, and pygmy chimpanzee), and four domestic animals (cattle, dog, goat, and horse). An interesting discovery in our study is that miR-197 does not regulate human *ADAM10*–3′UTR through the conventional miRNA seed sequence. Rather, miR-197 binds to an unconventional site on *ADAM10*-3′UTR via its 3′ side. The fact that this unconventional binding site but not the predicted miR-197 seed sequence is conserved between human and mouse suggests that the interaction between miR-197 to *ADAM10*-3′UTR is more similar to a siRNA. Moreover, although there is no conserved miR-197 in mouse, we identified a ncRNA Gm28836 in mouse, which performs similar function between MeCP2 and ADAM10. The effect of Gm28836 on mouse ADAM10 can be blocked by i-197 (Fig. 3m). These experiments further support that miR-197 regulating ADAM10 mRNA in a non-canonical way. As ADAM10 is not only involved in NPCs differentiation [44] but also affecting the radial migration of new-born neurons [45], it is possible that miR-197 may also affect other aspects of cortex development, in which ADAM10 may also be involved. Further investigations will be needed to fully understand the function of miR-197 in cortex development in primates.

Previous studies have shown that MeCP2 can downregulate and upregulate hundreds of different miRNAs in same samples [12, 13, 46]. Cheng et al.’s showed that MeCP2 blocks the biogenesis of miR-134 via interacting with DGCR8 [12]. However, there is also other study showed that MeCP2 can upregulate the biogenesis of miRNA, such as miR-199a, by interacting with DDX5 and Drosha [13]. In our system, we observed that MeCP2 still downregulates the biogenesis of miR-134 (Figure S5A) while it upregulates miR-197. These results suggested that the downregulation and upregulation mechanisms are not competing with each other. Tsujimura et al. showed that MeCP2 binds to pri-miR-199a [13]. Here we observed that MeCP2 could also bind to pri-miR-197 but not pri-miR-134 (Figure S5B). In addition, we also observed that overexpression of MeCP2 promoted the interaction of Drosha to pri-miR-197 (Figure S5C), but not pri-miR-134 (Figure S5D). Taken together, it is possible that the interaction of pri-miRNAs may affect the role of MeCP2 during miRNA biogenesis. That is to say, when MeCP2 binds to a pri-miRNA, it may facilitate the miRNA processing; but when MeCP2 does not bind to a pri-miRNA, it may interfere miRNA processing. It will be interesting to see whether other miRNAs regulated by MeCP2 also fit such rules.

Duplication or mutations of the *MECP2* gene has been shown to cause MDS and RTT, respectively. 100% of MDS patients and >60% of RTT patients express autistic behaviors, yet the underlying mechanism remains elusive. It has been proposed that altered NSC/NPC differentiation may contribute to the etiology of ASD [47]. However, the effect of *Mecp2* deficiency on NPCs fate in mouse was not all in agreement. There are couple of reports stated that *Mecp2* deficiency does not affect NPCs fate in the *Mecp2-/y* mouse embryonic cortex [48, 49]. However, several other reports showed that MeCP2 KO mice have reduced neuronal differentiation [50, 51] and iPSCs from RTT patients also have reduced neurogenesis [31]. Such different conclusions may due to different observation windows and methods in different studies. One possibility is that *Mecp2* deficiency may affect a sub-population of neurons in vivo as we observed Satb2^+^ neurons was increased in E18.5 and P7 Tg(*MECP2*) mouse brains (Fig. 1). Our observation on neonatal mouse cortex is also consistent with the clinical features of MDS patients, who have severe phenotypes starting from very early infantile stages [4, 5]. On the other side, most of the ASD cases in our study inherited the *MECP2* mutation from their asymptomatic carrier mother (Table 1). As all those ASD patients are boys, mutated *MECP2* on the X chromosome would affect them more severely than their mothers. As we observed strong loss-of-function effects of these rare *MECP2* mutations on neurogenesis, it is likely these *MECP2* mutations contribute to the etiology of ASD. To be noticed, the H371R mutation was identified in two unrelated cases in our ASD cohort (2/288), but does not exists in 61,075 controls from both ExAC and our cohort. Therefore, it is highly possible that H371R is a loss-of-function mutation with a high likelihood of being causative for ASD. What’s more, both our study and others have suggested that MeCP2-regulated miRNAs have the important functions in neurogenesis, including miR-197 and miR-199a [13, 31], it would be interesting to investigate the genetic variations of these key miRNAs in RTT and ASD patients in the future.

In conclusion, we uncovered a novel mechanism by which MeCP2 regulates NSC/NPC differentiation and miR-197 is a critical molecule downstream of MeCP2, which potentially could be important to the etiology of MDS and ASD.

## Materials and methods

### Animal housing and genotyping

Both C57BL/6 mice and FVB-Tg(*MECP2*)1Hzo/J mice were maintained in the animal facility at the Institute of Developmental Biology & Molecular Medicine, Fudan University. The protocol was approved by the Committee on the Ethics of Animal Experiments of Fudan University. The genotyping of FVB-Tg(*MECP2*)1Hzo/J mice were determined by PCR following the protocols from Jackson Laboratory website. https://www2.jax.org/protocolsdb/f?p=116:5:0::NO:5:P5_MASTER_PROTOCOL_ID,P5_JRS_CODE:14245,008679.

### Immunostaining on mouse cortical sections

For quantification of cell fate in WT and FVB-Tg(*MECP2*)1Hzo/J mice at E18.5 and P7, the regions of the primary somatosensory cortex were identified and the numbers of Sox2^+^, Tbr2^+^, or Satb2^+^ cells were counted in each vertical column with 100 μm width. All quantifications were performed with 4 brain sections from at least three animals. Data are presented as the mean ± SEM and statistical significance was assessed using unpaired Student’s *t*-test.

### Primary mouse NPCs isolation and differentiation assay

NPCs were isolated from E12.5–14.5 C57BL/6 or FVB mouse embryonic cortex. NPCs isolation and culture methods are based on previous reports [52, 53]. NPCs were either transfected with X-tremeGENE HP DNA transfection reagent (Roche, 06366236001) or infected with different lentivirus (Obio Technology (Shanghai) Corp., Ltd.). Seventy-two hours post-transfection or infection, NPCs cells were either lysed for western blot or subjected to immunofluorescent staining and imaging with a Zeiss LSM700 microscope. All films from western blot were scanned and analyzed with Quantity ONE based on intensity or were directly measured with Tanon gel image software. The results were normalized to its corresponding loading control GAPDH.

### miRNA inhibitors and mimics

Plasmids for hsa-miR-197 precursor (HmiR0013-MR04), hsa-miR-197 inhibitor (HmiR-AN0287-AM01), and scrambled negative control clone (CmiR0001-MR04) were purchased from GeneCopoeia, Inc. USA. The lentivirus for miR-197 (H4976) and miR-control (H32), i-197 (Y3496) and i-control (Y008) were all purchased from Obio Technology (Shanghai) Corp., Ltd. MiR-197 mimics (B01001) and scrambles (B04001); MiRNA inhibitors (B03001) and their negative controls (B04003) were purchased from Shanghai GenePharma Co, Ltd.

### Antibodies

Antibodies used in this study were as follow: from Abcam: ADAM10 (ab1997), ADAM17 (ab13535), NICD (ab8925), GFAP (ab7260), NOTCH1 (ab27526), DLL-1 (ab76655), JAG-1 (ab7771), Nestin (ab6142), MAP2 (ab11268, ab32454), GFAP (ab7260), Satb2 (ab92446), SOX2 (ab97959), TBR2 (ab23345), HA (ab9110); and Alexa Fluor® 647-labeled goat anti-Rabbit (ab150079), Alexa Fluor® 647-labeled goat anti-mouse (ab150115), or Alexa Fluor® 488-labeled goat anti-Rabbit (ab150077), Alexa Fluor® 488-labeled goat anti-mouse (ab150117). Anti-MeCP2 and GAPDH antibody was purchased from Cell Signaling (CST, #3456 & #2118S). All HRP-conjugated secondary antibodies were purchased from KangChen Bio-tech Inc., Shanghai, China.

### Plasmids and lentivirus

Human *MECP2*-e1 cDNA was purchased from Origene (RC202382). Point mutations were generated by mutagenesis PCR. The lentivrus for WT MeCP2 (HQ664), H371K (H4971), G428S (H4972), 380× (H4973), sh-MeCP2 (HQ363) and sh-control (H101) were all purchased from Obio Technology (Shanghai) Corp., Ltd. The corresponding mutations were also constructed into rat *MECP2*-e2 cDNA with a HA tag in pRK5 vector and used in IUE. The expression plasmid for mouse Gm28836 (GenBank ID XR_373236.2) (H11185) was also purchased from Obio Technology (Shanghai) Corp., Ltd.

Two different regions of human *ADAM10* 3′-UTR were cloned into XbaI/FseI sites of pGL3 plasmid to generate luciferase reporter constructs. A10-I-WT contains 1461-1765 of the *ADAM10* 3′-UTR, which covers the predicted miR-197 binding site (1568-1574) by TargetscanHuman (http://www.targetscan.org/vert_71/) [38]. A10-II-WT contains 4-405 of human *ADAM10* 3′-UTR, which covers the predicted binding site (186-201) by RNAhybrid analysis [39].

### qRT-PCR

Total RNA from NPCs, U251 or N2a cells was extracted with RNAeasy kit (QIAGEN, Cat# 74104). The levels of ADAM10 mRNA were determined using the qRT-PCR kit (Takara RR036A and RR820A) with *GAPDH* as internal control. The levels of mature miR-197 were determined with All-in-One™ miRNA qRT-PCR Detection Kit (GeneCopoeia, QP015). The levels of pri-miR-197 were detected with TaqMan pri-microRNA assay kit (Applied Biosystems) following reverse transcript with Toyobo ReverTra Ace-α kit (Toyobo). The levels of pre-miR-197 were detected with miScript Precusor Assay (QIAGEN) followingreverse transcript with miScript II RT Kit (QIAGEN).

### RNA immunoprecipitation

U251 cells were transfected with empty vector, WT MeCP2, or ASD-related MeCP2 mutant expressing plasmids. Twenty-four hours later, cells were collected for cross-linking, lysing, and sonication and were subjected to the RNA immunoprecipitation assay using previously described protocols [13, 54]. Anti-MeCP2 antibody (ab2828) and rabbit immunoglobulin G (IgG) antibodies were used. The immunoprecipitated RNA was analyzed by qRT-PCR described before.

### Biotinylated micro-RNA pull down assay for identifying miRNA targets

Biotinylated double stranded miRNA-197 and its scrambled control miRNA (B02003) (both Biotin-labeled at 3′ end) were purchased from GenePharma. U251 cells were transfected with control miRNA and miR-197 at a final concentration of 100 nM with RNAimax (Invitrogen^TM^, 13778150). Twenty-four hours post-transfection, whole cell lysates were harvested and subjected to RNP pull-down followed with RT-PCR. The miRNA enrichment was calculated as follow: Bi-miR-197 pull-down for ADAM10 3′UTR/Scramble control pull-down for ADAM10 3′UTR = X, Bi-miR-197 input/Bio-Scramble control input = Y, Fold binding = X/Y. At least three independent experiments with a minimum of three replicates each time were performed for each set.

### In utero electroporation and cell counting

In utero electroporation was performed as previously described [55, 56]. About 1.5 μl of DNA mix was injected into each embryo. The ratio of either expressing plasmid or empty vector to pEYFP was 6:1. The ratio of (miR-197 inhibitor or ADAM10): (WT MeCP2): pEYFP was 3:3:1. The ratio of miR-197, MeCP2^G428S^, and pEYFP was 3:3:1. After electroporation and recovery, E18.5 embryos were collected and sectioned at 20μm and processed for further immunofluorescent analyses. Nuclear cell staining with DAPI was used to define different sub-regions of the cerebral cortex based on cell density, as previously described [55]. The percentage of EYFP^+^ cells in each layer was calculated based on total number of EYFP^+^ cells in the same brain section. At least three sections from one brain were collected and at least four different embryos obtained from 3 to 4 different pregnant dams were collected for each group for statistical analyses.

### Statistical analyses

All experiments were repeated at least three times and the statistical significance was evaluated. Data are expressed as mean ± SEM. Statistical differences were calculated by two-tailed unpaired *t*-test for two datasets and ANOVA followed by Bonferroni post-hoc test for multiple datasets using Prism (GraphPad Inc., La Jolla, CA). *p* < 0.05 was considered statistically significant.

### Human subjects

Blood samples from 288 ASD patients (mean age 6.1 ± 3.1 years, 85% male) were collected between 2007 and 2010 at the Shanghai Mental Health Center of Shanghai Jiaotong University. Protocols were reviewed and approved by the Ethics Committee of Fudan University and Shanghai Jiaotong University prior to the commencement of the study. Written informed consent from the parents or guardians of the children was obtained prior to inclusion in the study. The 369 controls (mean age 19 years, 81% male) were unrelated healthy volunteers from the freshman student class at Fudan University (2010), which were ethnically and gender-matched from the same geographical area. DNA was extracted and all samples were pooled together for targeted sequencing on the exons of *MECP2* gene was carried out at GBP Biotechnology (Jiangsu, China). Sanger sequencing was performed to confirm those 5 missense mutations in the 6 ASD cases (Fig. S4A).

## Supplementary information

Supplementary
