# Supplementary material for: MicroRNA-197 controls ADAM10 expression to mediate MeCP2’s role in the differentiation of neuronal progenitors
Source: Cell Death Differ. 2018 Dec 18;26(10):1863–79. doi: 10.1038/s41418-018-0257-6 (PMC6748079; doi:10.1038/s41418-018-0257-6)
Supplement: Supplementary file 1 — Supplementary [file 41418_2018_257_MOESM1_ESM.docx]

**MicroRNA-197 controls ADAM10 expression to mediate MeCP2’s role in the differentiation of neuronal progenitors**

Yu-Meng Wang^2,1,#^, Yu-Fang Zheng^1,2,3,#,^*, Si-Yu Yang^2,1^, Zhang-Min Yang^4^, Lin-Na Zhang^5^, Yan-Qin He^4^, Xiao-Hong Gong^1^, Dong Liu^6^, Richard H. Finnell^7^, Zi-Long Qiu^8^, Ya-Song Du^5,^*, Hong-Yan Wang^1,3,9,^*

1. Obstetrics and Gynecology Hospital, State Key Laboratory of Genetic Engineering at School of Life Sciences, Institute of Reproduction and Development, Fudan University, Shanghai 200011, China
2. Institute of Developmental Biology & Molecular Medicine, Fudan University, Shanghai 200433, China
3. Key Laboratory of Reproduction Regulation of NPFPC, Collaborative Innovation Center of Genetics and Development, Fudan University, Shanghai 200032, China
4. Department of Biochemistry and Molecular Biology, College of life Sciences, Shaanxi Normal University, Xi’an 710062, China
5. Shanghai Mental Health Center, Shanghai Jiaotong University, Shanghai 200030, China
6. Co-innovation Center of Neuroregeneration, Jiangsu Key Laboratory of Neuroregeneration, Nantong University, Nantong, Jiangsu 226001, China
7. Departments of Molecular and Cellular Biology and Medicine, Baylor College of Medicine, Houston, Texas 77030, USA, and Collaborative Innovation Center for Genetics & Development, School of Life Sciences, Fudan University, Shanghai 200438, China
8. Institute of Neuroscience, Shanghai Institutes for Biological Sciences, Chinese Academy of Sciences, Shanghai, 200031, China
9. Children’s Hospital of Fudan University, 399 Wanyuan Road, Shanghai 201102, China and Institutes of Biomedical Sciences, Fudan University, Shanghai 200032, China

# These authors contribute equally.

* Corresponding authors: Hongyan Wang: wanghy@fudan.edu.cn; Yasong Du: 13501942224@163.com; Yufang Zheng: zhengyf@fudan.edu.cn

**Key words:** MECP2, MDS, miR-197, ADAM10, neural progenitor cells

**Running title:** MeCP2 regulates NPCs differentiation via miR-197

**Supplementary material and methods**

**Primers**

The primers for hsa-mir-197-3p (HmiRQP0287) and the internal control snRNA U6 gene (HmiRQP9001 for human U6 and MmiRQP9002 for mouse U6) were also purchased from GeneCopoeia Inc. Primers for pri-miR-197 (4427012), pri-miR-134 (4427012), and the internal control GAPDH gene (4331182) were purchased from Applied Biosystems. Primers for pre-miR-197 (Hs_mir-197_PR_1 miScript Precursor Assay, MP00001302), pre-miR-134 (Hs_mir-134_PR_1 miScript Precursor Assay, MP00000847) and the internal control U6 gene (Hs_RNU6-2-11 miScript Primer Assay, MS00033740) were purchased from QIAGEN.

Point mutations in MECP2 were generated with the following primers.

H371R: 5’-GAGCACCACCACCATCACCGCCACTCAGAGTCCCCAAAG-3’,

5’-CTTTGGGGACTCTGAGTGGCGGTGATGGTGGTGGTGCTC-3’;

E394K: 5’-CCACCTGAGCCCAAGAGCTCCGAGG-3’

5’-CCTCGGAGCTCTTGGGCTCAGGTGG-3’;

G428S: 5’-CACTGGAGAGCGACAGCTGCCCCAAGGAGCC-3’

5’-GGCTCCTTGGGGCAGCTGTCGCTCTCCAGTG-3’

The primers used for PCR cloning A10-II-WT are as follow.

F: 5’-CAGTCTAGACAGCTTTTGCCTTGGTTCTT-3’;

R: 5’-ACTGGCCGGCCGGTCGAGCCTCCTAGCCTTGATTG-3’.

The primers used to generate A10-II-Mut are as follow:

F1: 5’-CAGTCTAGACAGCTTTTGCCTTGGTTCTT-3’;

R1: 5’-AAGAAAATTGGGTTCCTTTTAATTGGA ATTTTCAGGCTTT-3’;

F2: 5’-AAAGCCTGAAAATTCCAATTAAAAGGAACCCAATTTTCTT-3’;

R2: 5’-ACTGGCCGGCCGGTCGAGCCTCCTAGCCTGATTG-3’.

The primers used for RT-PCR hADAM10 3’UTR in Biotinylated Micro-RNA Pull Down Assay were:

5’-CAGTCTAGACAGCTTTTGCCTTGGTTCTT-3’;

5’-ACTGGCCGGCCGGTCGAGCCTCCTAGCCTTGATTG-3’

The primers used for qRT-PCR were:

Human ADAM10: F: 5’- TCCCCTTGCAACGATTTTAGAG-3’;

R: 5’- AGGAGGAGGCAACTTTGGATTACT-3’;

Human β-Actin: F: 5’- GGGAAATCGTGCGTGACATTAAG -3’

R: 5’- TGTGTTGGCGTACAGGTCTTTG -3’

Mouse Adam10: F: 5’-CCTGCCATTTCACTCTGTCATTTA-3’

R: 5’-GTGCCCGGGCTCCTTCCTCTACTC-3’;

Mouse Gapdh: F: 5’- ACAGCAACTCCCACTCTTCCACCT -3’

R: 5’- TTGCTCAGTGTCCTTGCTGGGG -3’

The primers used for RT-PCR to detect the mouse transcripts in figure S3E

| Location/transcript | Primers | Product length |
| --- | --- | --- |
| Chr. X:139790709-139790884 | F: 5’-AGCCTGAATGCACAGTGAAA-3’  R: 5’-TCGCTCCTCTTTCTGAGC-3’ | 80 bp |
| Chr. 2:171933205-171933220 | F: 5’- ATGTTTTAAACTTAGCGACCTACA -3’  R: 5’- CTTCAAAATCCCACCCCTAACAAT -3’ | 72 bp |
| Chr. 5:87639674-87639829 | F: 5’- CCACAGCCTCCTGTTTTTGC -3’  R: 5’- TGGGGAATGCAGTACGTGTC -3’ | 76 bp |
| Gm16196 | F: 5’- GTGATCATTGCTAAGGCACCAG -3’  R: 5’- TTTCTTTCCACTGAAAAACACCT -3’ | 97bp |
| Gm17296 | F: 5’- CAGGGCTTTGGCAAGCATAC -3’  R: 5’- CGCTGGAGAACATCCCTGAG -3’ | 78bp |
| Gm41705 | F: 5’- TTGGGTGCCTGTCTCTGTTC -3’  R: 5’- AACGTGCCTATGGCCAGC -3’ | 74bp |
| Gm28836-1 | F: 5’-CCAGACCTATGCTCCGACAC-3’  R: 5’-GCCAGCAGAGGCCAAGAAG-3’ | 57 bp |
| Gm28836-2 | F: 5’- AGACTCCAGACCTATGCTCC -3’  R: 5’- TGACCCGCCTTAAGTGTTCTC -3’ | 196bp |
| Gm28836-3 | F: 5’- GCAGACTCCAGACCTATGCTC -3’  R: 5’- GGAGTTGGTTTGTGGGAGGT -3’ | 144bp |
| Gm28836-4 | F: 5’- AGCAGACTCCAGACCTATGC -3’  R: 5’- TGTGACCCGCCTTAAGTGTT -3’ | 202bp |
| Gm33911 | F: 5’-CAGGTCTCCTGAACTTGTAAC-3’  R: 5’-AGGTATTGGATCGTCAGTAACA-3’ | 80 bp |
| Mouse β-Actin | F: 5’- GAGACCTTCAACACCCCAGC -3’  R: 5’- ATGTCACGCACGATTTCCC -3’ | 263bp |
| Mouse Gapdh | F: 5’- ACAGCAACTCCCACTCTTCCACCT -3’  R: 5’- TTGCTCAGTGTCCTTGCTGGGG -3’ | 189bp |

**The sequences of inhibitors of miRNA used in this study**

hsa-miR-197-3p, 5’-GCUGGGUGGAGAAGGUGGUGAA-3’;

hsa-miR-221-3p, 5’-GAAACCCAGCAGACAAUGUAGCU-3’;

hsa-miR-199a-5p, 5’-GAACAGGUAGUCUGAACACUGGG-3’,

hsa-miR-222-3p, 5’-ACCCAGUAGCCAGAUGUAGCU-3’,

hsa-miR-137, 5’-CUACGCGUAUUCUUAAGCAAUAA-3’,

hsa-miR-193a-3p, 5’-ACUGGGACUUUGUAGGCCAGUU-3’,

hsa-miR-184, 5’-ACCCUUAUCAGUUCUCCGUCCA-3’,

hsa-miR-187-3p, 5’-CCGGCUGCAACACAAGACACGA-3’.

**

**

**Figure S1. *MECP2* duplication caused more neurogenesis in NPCs isolated from Tg*(MECP2)* mice brain.** Mouse primary NPCs isolated from FVB WT and Tg(*MECP2*) mouse E12.5 embryonic cortex were directly cultured for 72hrs. Cell lysates were subjected to western blot analysis for both MeCP2 and differentiation markers, MAP2 and GFAP. GAPDH was used as loading control. N=3. Representative blot is shown in left and statistical analysis for MAP2, GFAP, and MeCP2 levels are shown in right panels, respectively. All statistic data represent means ± SEM. * *p*<0.05, ** *p*<0.01, *** *p*<0.001.

**
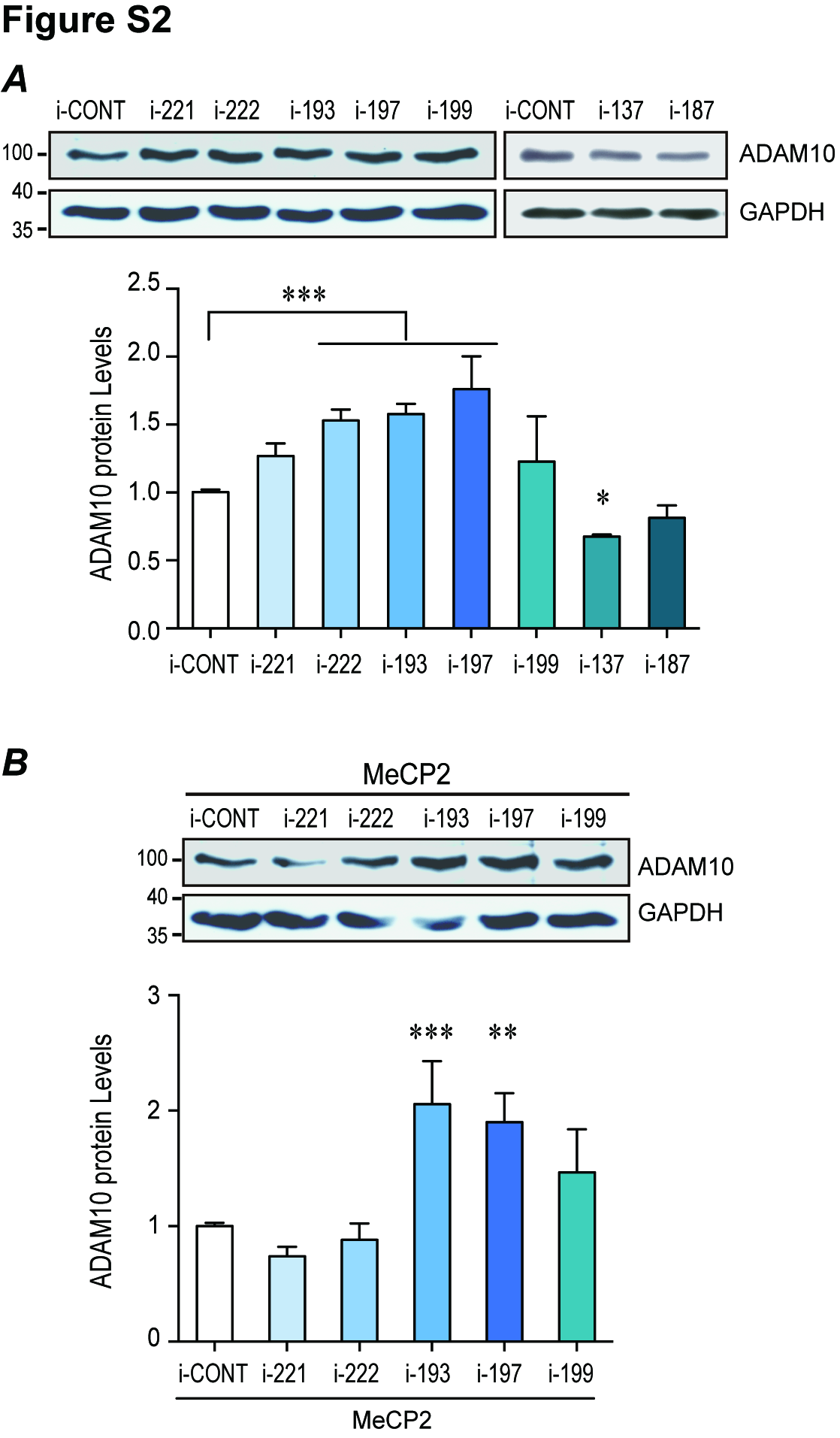
**

**Figure S2. MiR-197 inhibitor reversed the down-regulation effect of ADAM10 by MeCP2. (A)** Inhibitors for miR-221, miR-222, miR-193, miR-197, miR-199, miR-137, and miR-187 were transfected into U251 cells and the levels of ADAM10 protein were examined. **(B)** Inhibitors for miR-221, miR-222, miR-193, miR-197, and miR-199 were co-transfected with WT MeCP2 into U251 cells, and the levels of ADAM10 protein were examined. Representative blots are shown on the top panels, and statistical analyses for ADAM10 protein are shown on the lower panels. All data represent means ± SEM. N≥3, * *p*<0.05, ** *p*<0.01, *** *p*<0.001.

**
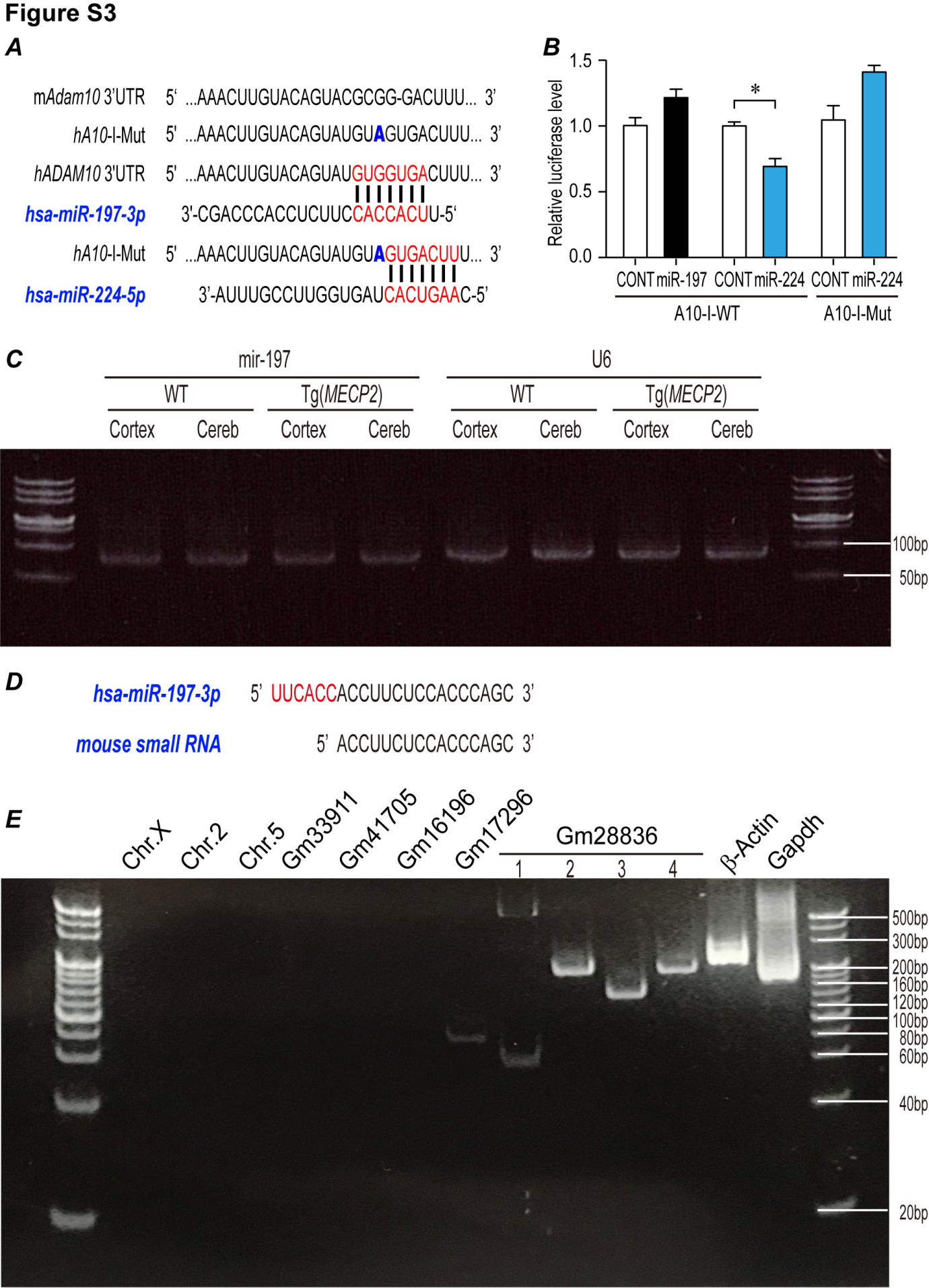
**

**Figure S3. A miR-197 like small RNA could be RT-PCR amplified from E18.5 mouse cortex. (A)** A 7mer-m8 miR-197 binding site at position 1568-1574 of human *ADAM10* 3’-UTR was predicted by TargetscanHuman, which is poorly conserved between human and mouse. The alignment of miR-197 to human *ADAM10* 3’-UTR and miR-224 to A10-I-Mut were illustrated. **(B)** Luciferase reporter assay showed that miR-224 but not miR-197 could down-regulate the expression of A10-I-WT. The point mutation A10-I-Mut is not sensitive to miR-224 anymore. All data represent means ± SEM. N≥3, * *p*<0.05. **(C)** Fetal cortex and cerebellum from E18.5 WT and Tg(*MECP2*) mice were dissected out. RNA was extracted and subjected to RT-PCR with either hsa-miR-197 specific primer or control Rnu6 (U6 small nuclear RNA) primers. 8μl products and the DNA ladder DL500 (Takara) were run on 12% non-denaturing PAGE gel without urea and stained with Gel-Red for 40min. **(D)** The PCR product was cloned into T-vector and sent for sequencing. The small RNA from mouse brain has 16 identical nucleotides to the 3’ side of has-miR-197. **(E)** Fetal cortex from E18.5 WT mice were dissected out. RNA was extracted and reversely transcripted to cDNA with ReverTra Ace® qPCR RT Master Mix with gDNA Remover (TOYOBO, #FSQ-301). Then the cDNA was subjected to PCR with primers designed for different loci and transcripts. Three genomic loci, four ncRNAs (Gm33911, Gm41705, Gm16196, Gm28836) and one mRNA (Gm17296) were tested. Four sets of different primers were used for Gm28836. β-Actin and Gapdh were used as positive control. 8μl products and the DNA ladder DL3420A (Takara) were run on 12% non-denaturing PAGE gel without urea and stained with Gel-Red for 40min.

**
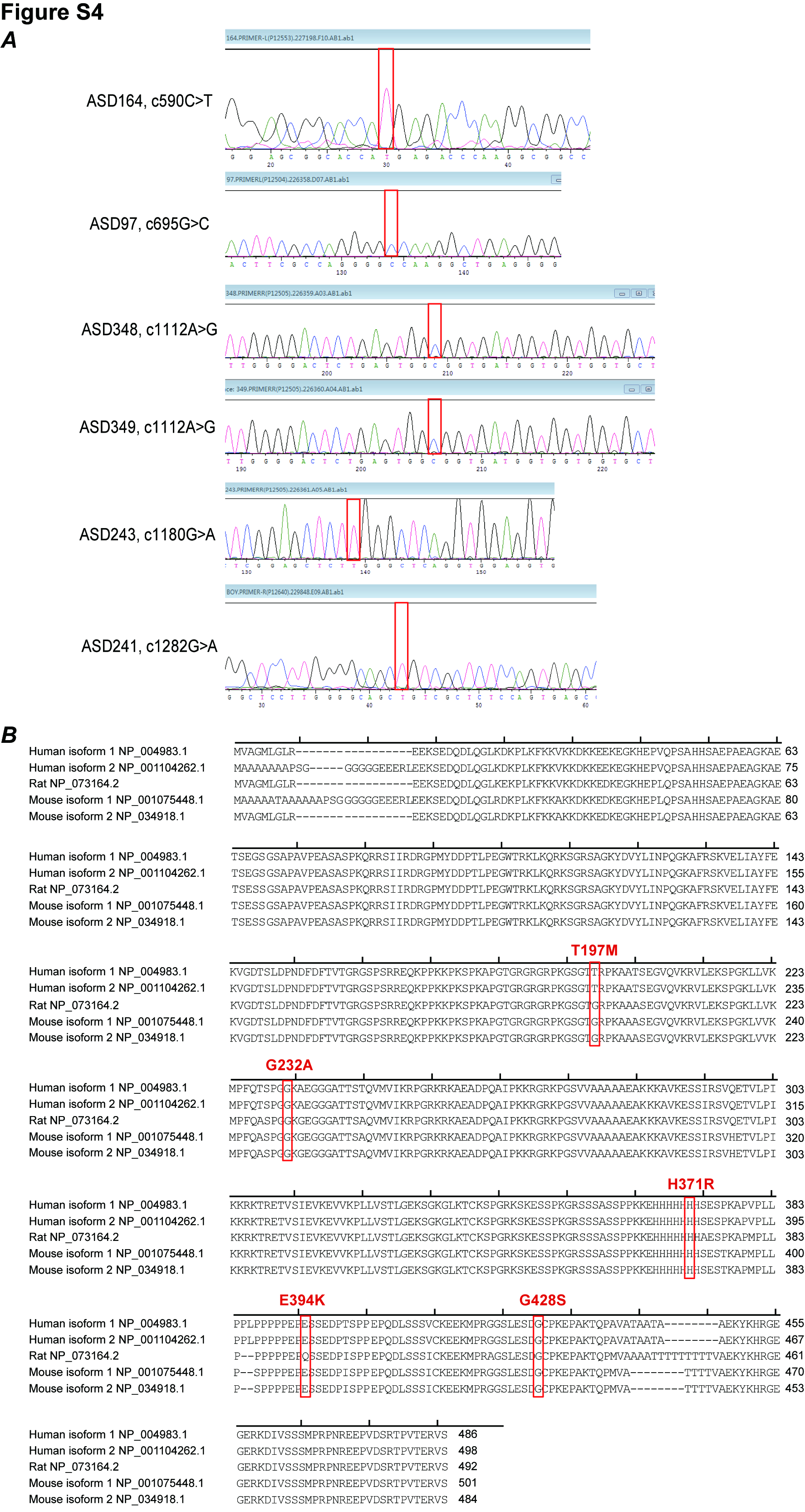
**

**Figure S4. The sequencing data and alignment of autism-related MECP2 mutations. (A)** The MECP2 mutations in the ASD patients were confirmed by Sanger sequencing. **(B)** MeCP2 is a highly conserved protein. Sequence alignment of human MeCP2 e1 and e2, mouse MeCP2 e1 and e2, and rat MeCP2 e2 proteins by NCBI online alignment software. The mutated amino acids identified in ASD cases are highlighted in red boxes.

**
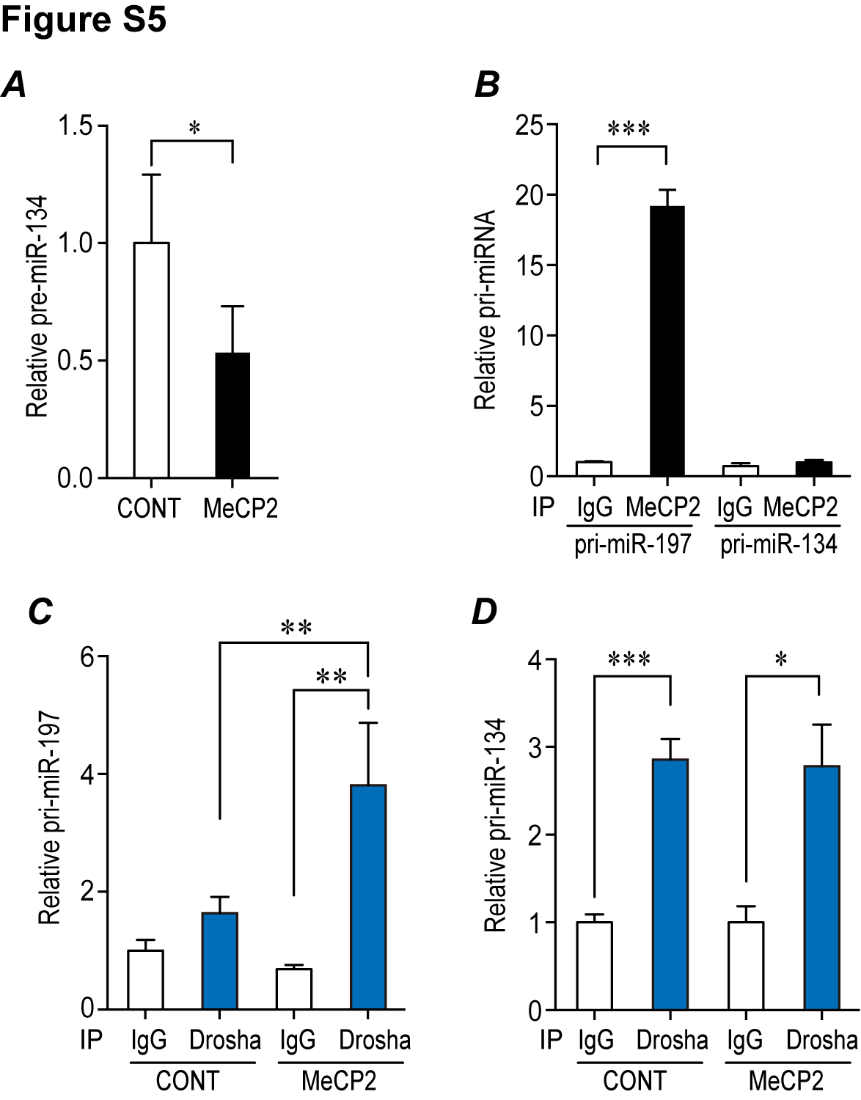
**

**Figure S5. MeCP2 interacts to pri-miR-197 but not pri-miR-134 and still down-regulates pre-miR-134. (A)** U251 cells transfected with MeCP2 expressing plasmids were subjected to qRT-PCR for pre-miR-134 (N=4) **(B)** U251 cells transfected with MeCP2 expressing plasmids were subjected to RNA-IP with MeCP2 antibody or control IgG. The levels of pri-miR-197 and pri-miR-134 co-precipitated with MeCP2 were quantified by qRT-PCR (N=5). **(C-D)** U251 cells transfected with control vector or MeCP2 expressing plasmids were subjected to RNA-IP with Drosha antibody or control IgG. The levels of pri-miR-197 **(C)** and pri-miR-134 **(D)** co-precipitated with Drosha were quantified by qRT-PCR (N=3). All statistical data represent means ± SEM. * *p*<0.05, ** *p*<0.01, *** *p*<0.001.

**Table S1. The top aligned mouse ncRNA transcripts and genomic sequences by blasting 16nt 5’-ACCUUCUCCACCCAGC-3’ (data extracted from the NCBI BLAST database).**

|  | Description | Max score | Query cover | E value | Accession Number | Length (bp)*  /position^#^ |
| --- | --- | --- | --- | --- | --- | --- |
| Transcripts | Mus musculus predicted gene 16196 (Gm16196), transcript variant X6, **ncRNA** | 30.2 bits (15) | 93% | 5.0 | XR_001783002.1 | 734 |
|  | Mus musculus predicted gene, 41705 (Gm41705), **ncRNA** | 30.2 bits (15) | 93% | 5.0 | XR_877341.2 | 14410 |
|  | Mus musculus predicted gene 16196 (Gm16196), transcript variant X3, **ncRNA** | 30.2 bits (15) | 93% | 5.0 | XR_876717.1 | 1134 |
|  | Mus musculus predicted gene 28836 (Gm28836), **ncRNA** | 30.2 bits (15) | 93% | 5.0 | XR_373236.2 | 1436 |
|  | Mus musculus predicted gene, 33911 (Gm33911), **ncRNA** | 28.2 bits (14) | 87% | 20 | XR_389366.2 | 1338 |
| Genomic sequences | Mus musculus strain C57BL/6J chromosome 2, | 32.2 bits (16) | 100% | 1.3 | NC_000068.7 | 171933205 to 171933220 |
|  | Mus musculus strain C57BL/6J chromosome 5, | 32.2 bits (16) | 100% | 1.3 | NC_000071.6 | 87639744 to 87639759 |
|  | Mus musculus strain C57BL/6J chromosome X, | 32.2 bits (16) | 100% | 1.3 | NC_000086.7 | 139790779 to 139790794 |

* length of the transcript

# the position of the matched sequence within the correspondence locus.
